# Factors Associated with Utilization of Complete Postnatal Care Service in Baglung Municipality, Nepal

**DOI:** 10.1155/2020/2892751

**Published:** 2020-07-19

**Authors:** Sita Chhetri, Rajani Shah, Laxmi Rajbanshi

**Affiliations:** ^1^Charak Hospital Nursing College, Pokhara, Kaski, Nepal; ^2^Nepal Public Health Foundation, Kathmandu, Nepal; ^3^Teaching Hospital, Chitwan Medical College, Bharatpur, Nepal

## Abstract

**Background:**

Postnatal period is six weeks after birth. It is critical but is the most neglected period. A large proportion of maternal and neonatal deaths occur during 48 hours following childbirth. The utilization of the recommended three postnatal checkups within seven days after delivery, which plays a vital role in preventing maternal and neonatal deaths, is low in Nepal.

**Objective:**

This study is aimed at identifying the factors associated with the utilization of complete postnatal care (PNC) among mothers.

**Method:**

A cross-sectional study was carried out among 318 mothers in wards 1, 2, 3, and 4 of Baglung municipality, Nepal. Data was collected by semi-structured interviews. Descriptive analysis and comparison of characteristics of women/families with complete vs. partial postnatal checkups using multivariable logistic regression were done.

**Results:**

Among 314 respondents receiving at least one PNC, 78% had partial and 22% had complete PNC. Relatively advantaged caste/ethnicity- Brahman/Chhetri (aOR = 3.18, 95% CI: 1.24-8.12) and Janajati (aOR = 2.87, 95% CI: 1.09-7.53) - compared to Dalits, husbands working as a job holder in Nepal (aOR = 3.49, 95% CI: 1.50-8.13), and delivery in a private hospital (aOR = 11.4, 95% CI: 5.40-24.2) were associated with having complete PNC.

**Conclusion:**

Although PNC attendance at least once was high, utilization of complete PNC was low. More focus to mothers from disadvantaged caste/ethnicity, those whose husbands are in foreign employment, and improvement in quality of care in government health facilities may increase the use of complete PNC.

## 1. Introduction

A large proportion of maternal and neonatal deaths occur during 48 hours following childbirth. Almost all (99%) newborn and maternal deaths happen in developing countries. Globally, there were an estimated 303000 maternal deaths from complications related to pregnancy and childbirth in 2015 [1]. South Asia and Sub-Saharan Africa shared the highest proportion [2]. The first week of birth accounts for the highest number of the deaths [3]. In Nepal, maternal mortality ratio stands at 239 per 100000 live births, while neonatal mortality rate is 33 per 1000 live births [4]. Sustainable development goal aims at reducing the MMR to less than 70 per 100000 live births, reducing preventable deaths of newborn to less than 1%, and providing postnatal care (PNC) for 90 percent of mothers by 2030 [5].

Postnatal period is up to six weeks after childbirth. The major purposes of PNC are to maintain and promote the health of the woman and her baby. It is generally the most neglected period in developing countries. Mothers and newborn babies do not receive PNC from a skilled health care provider during the first few days after delivery [6]. The national essential maternal and neonatal health care service package for Nepal recommends three PNC for all women, whether delivered at home or at health facility: first PNC within 24 hours, second and third ones on the third and seventh day, respectively [7].

Nepal Demographic and Health Survey 2016 showed that only 57% of mothers received a PNC within two days of delivery [4]. According to the Department of Health Services Nepal, the proportion of mothers having three PNC visits as per protocol stands at 11% in Baglung district, compared to 18% at the national level [8]. The national figure even declined to 16% in 2018 from 19% in 2017. The low utilization of the service has been attributed to cultural and geographical factors as well as perceived low importance of care during the postpartum period [9]. Utilization of maternal health services is a complex phenomenon influenced by many factors. Sociodemographic factors such as age, education, wealth index, ethnicity, culture, decision making power, religion and service delivery environment are important determinants for the use of maternal health services [10, 11].

Although information on the use of PNC in Nepal is available [11–14], there is very little information about the use of the service according to protocol [9]. Hence, this study assessed the magnitude of and factors associated with the utilization of complete PNC service.

## 2. Methods

### 2.1. Study Area

Baglung district which represents a typical hilly district of Nepal and that has low utilization of PNC service was chosen [8]. It has population of 270000. The center of the district, i.e., Baglung municipality, is about 275 kilometer west of Kathmandu, the capital of Nepal, and is connected to Nepal's one of the biggest cities, Pokhara. According to district population profile 2017, the total population of Baglung municipality is 63511, with 1495 children of age 0-11 months [15]. Out of 14 wards of the municipality, ward numbers 1, 2, 3, and 4 are city areas of the municipality and are connected to one government hospital and two private hospitals located at headquarter of the municipality by concrete road. It takes less than 30 minutes to reach the hospitals by walking from wards 1, 2, and 4, whereas 10 to 60 minutes is needed from ward number 3 as it stretches from the market area to its outskirts.

### 2.2. Study Design and Population

A community based cross-sectional study was conducted among mothers residing in the selected wards of the municipality who gave birth a week before data collection to the last 12 months.

### 2.3. Sample

Sample size of the study was calculated by using the population proportion formula taking into account the proportion of women using PNC service (*p*) = 50% [16, 17], 95% confidence level, and margin of error (*d*) = 5.8%.

(1) $$\begin{array}{c} \text{Sample size }\left(n\right)=\frac{Z^{2}pq}{d^{2}}=\frac{\left(1.96\times 1.96\right)\left(0.05\right)\times \left(0.05\right)}{{\left(0.58\right)}^{2}}285.49 \end{array}$$

Considering a non-response rate of 10%, the final sample size of the study was 317.21. Hence, a total of 318 respondents were included in the study.

### 2.4. Sampling

Baglung municipality, the only municipality of the district, was purposively selected as the study aimed at assessing the use of PNC service in an urban setting where the service is relatively more accessible. Four connected wards, namely, ward numbers 1 to 4, were also purposively selected being the market area and its surrounding. All the mothers who had given birth one week prior to data collection for the past 12 months and residing in the selected wards of ward numbers 1, 2, and 3 and some mothers of ward number 4 were interviewed by house screening.

### 2.5. Data Collection and Procedure

Data was collected from 25^th^ June to 25^th^July 2017 by face-to-face interview using a pretested [18] semi-structured questionnaire in Nepali language. The questionnaire included questions on use of PNC service, socio-demographic variables (age, education and occupation of respondents, caste/ethnicity, annual family income, and occupation of husbands), obstetrical variables (parity, antenatal care (ANC), place of delivery, duration of hospital stay, and complication during delivery), and awareness of PNC service. The number of mothers was identified from the registry of female community health volunteers of the respective wards and immunization registry of the local health facility. Then, house-to-house visit was done for data collection. Data was collected by the first author. The collected data was edited on the same day throughout the data collection period.

### 2.6. Variables

#### 2.6.1. Dependent Variable

The dependent variable was the ‘utilization of complete PNC.'

#### 2.6.2. Independent Variables

Independent variables include socio-demographic characteristics, ANC visit, type of facility used for childbirth, complication during childbirth, duration of hospital stay, and awareness of PNC services.

### 2.7. Operational Definition

#### 2.7.1. Utilization of Complete PNC

Utilization of complete PNC refers to at least three PNC within seven days after delivery. It was classified into two categories based on frequency and timing of postnatal checkup: ‘partial' means one and/or two PNC within 24 hours and on third day following delivery, respectively, while ‘complete' means three PNC with at least the third one on the seventh day following delivery.

1. Caste/Ethnicity. ‘Brahman/Chhetri' (so-called upper caste group), ‘Janajati', and ‘Dalits' [19]
2. Education. Education was measured in terms of the ability to read and write and level of education completed. The four categories included ‘no schooling,' ‘basic education' (completion of any of the grades from 1 to 8), ‘secondary education' (completion of any of 9 to 12 grades), and ‘higher education' (bachelor's degree or higher) [20]
3. Economic Status. Total yearly income of the household in Nepalese rupee (NPR) was classified into three categories: ‘less than 250000,' ‘250000-600000,' and ‘more than 600000' [21, 22]
4. Parity. The parity of the recent birth was categorized as ‘one' and ‘two or more' for further analysis. Type of Family. The type of family was categorized as ‘nuclear' and ‘joint'
5. Number of ANC Visit. The number of ANC visit a respondent had during her last pregnancy was counted. The responses were grouped as ‘no visit,' ‘less than 4 visits,' and ‘4 or more visits'
6. Childbirth Complication. Complication that arose during last childbirth. It was categorized as ‘yes' and ‘no'
7. Type of Health Facility Used for Childbirth. ‘Government facility' and ‘private facility'
8. Duration of Hospital Stay after Delivery. The duration of hospital stay after delivery was categorized as ‘6 hours or less,' ‘7 to 24 hours,' and ‘more than 24 hours' [3, 7, 23].
9. Awareness of PNC Services. It refers to information regarding meaning of postnatal period, timing and frequency of PNC visit, recommended health care provider for PNC, types of PNC services, and danger signs that may arise during postnatal period. It was classified into two categories based on the mean score: ‘good awareness' (mean ≥ 5.50) and ‘poor awareness' (mean < 5.50)

### 2.8. Data Processing and Analysis

Collected data were checked daily for completeness, consistency, and accuracy. The data were coded and then entered in data entry software EpiData 3.1 [24] and analyzed in the software Statistical Package for the Social Sciences version 18 [25]. Descriptive and inferential statistics were used for data analysis. As descriptive statistics, frequency, percentage, mean and standard deviation were used. In inferential statistics, chi-square test was applied to find out the association between level of utilization of PNC and independent variables. The variables that showed significant association at 5% level of significance were further analyzed to estimate unadjusted and then adjusted odds ratio (aOR) using multivariable logistic regression [26] to determine the factors associated with the utilization of complete PNC and their strength of association.

### 2.9. Ethics and Consent to Participate

Ethical approval was obtained from Chitwan Medical College-Institutional Review Committee. Permission to carry out the study was taken from municipality office of Baglung. Verbal informed consent was obtained from each respondent by clarifying the purpose of the study prior to the data collection. Confidentiality was maintained by not disclosing the information to anyone else and using it for research purpose only. Respondents' dignity was maintained by giving the right to reject or discontinue from the study at any time without any penalty.

## 3. Results

### 3.1. Sociodemographic Characteristics of the Respondents

Among 318 total respondents, 39% were in the age group 20-24 years. Regarding caste/ethnicity, about 47% were Brahman/Chhetri. Around 91% of the respondents belonged to the Hindu by religion. About two-thirds (63.7%) of the respondents had secondary level schooling. With regard to occupation, more than half (55.2%) of the respondents were involved in household work. Regarding the type of family, 54% of the respondents belonged to a nuclear family. More than one-third (35.6%) of the respondents stated that the annual income of their family was 250000 to 600000 NPR. About 31% of the husbands were involved in foreign employment. Nearly half (48.7%) of the respondents had given birth for the first time (Table 1).

### 3.2. Obstetrical Characteristics of the Respondents

Nearly all (99.4%) of the respondents had ANC checkup during last pregnancy. Of these, around 88% had four or more ANC. Regarding the place of delivery, almost all (98.7%) of the respondents had their last delivery in a health facility, while 84% of these used government hospitals for delivery. Around three-quarters (74.8%) of the mothers delivered by spontaneous vaginal delivery, while about 22% of the respondents had caesarean section. Slightly more than two-thirds (67.2%) of the births were assisted by a staff nurse/auxiliary nurse midwife (ANM). Regarding duration of hospital stay after delivery, 50% of the respondents stayed for seven to 24 hours. About 19% of the mothers had complication during the last childbirth. Half of the respondents (50.6%) had poor level of awareness on PNC services (Table 2).

### 3.3. Utilization of PNC

Of the total respondents, almost all (98.7%) had PNC. Among them, 78% had partial utilization (1-2 visit), while 22% had complete PNC. About 62% of the respondents received the PNC from nurses, and 85% of the respondents received the service from government hospital (Table 3).

### 3.4. Factors Associated with Utilization of Complete PNC

Caste/ethnicity, occupation of respondent's husband, type of health facility used for delivery, complication during delivery, duration of hospital stay after delivery, and awareness level on PNC service were found to be significantly associated with the utilization of complete PNC (Table 4).

### 3.5. Odds Ratio of Factors Associated with Use of Complete PNC

In multivariable logistic regression analysis, only caste/ethnicity, occupation of respondent's husband, and type of health facility where delivery happened were significantly associated with the utilization of complete PNC. The mothers who belonged to Brahman/Chhetri (aOR = 3.18, 95% CI: 1.24-8.12) and Janajati (aOR = 2.87, 95% CI: 1.09-7.53) caste/ethnicity were more likely to have complete PNC as compared to Dalits. Similarly, respondents whose husbands were job holders within the country were more likely (aOR = 3.49, 95% CI: 1.50-8.13) to get complete PNC as compared to those whose husbands were in foreign employment. Respondents who delivered their last child at a private hospital had higher likelihood (aOR =11.4, 95% CI: 5.40-24.2) of having complete PNC compared to those who delivered in a government hospital (Table 5).

## 4. Discussion

The study showed that about 99% of the respondents had utilized PNC service at least once, 34.7% had received two PNC, and only 21.7% of them had received the service three times within seven days following delivery. A similar study carried out in India found that 36.6% of the respondents had received one postnatal checkup, 18.5% received two, and 14.7% had three postnatal checkups [27]. Likewise, a study conducted in Ethiopia revealed that 57.4% of the mothers had one PNC, 40.4% had two PNC, and 2.2% had three or more PNC [28]. The difference in the proportions in the present and the previous studies may be attributed to the study setting and study population. Regarding the level of the utilization of PNC, 78% of the respondents had partial (1 to 2 times) utilization, while only 22% of the respondents had utilized complete PNC. A national figure showed that in Nepal 19% of women had PNC checkup as per protocol [9]. Similarly, a study conducted in Myanmar demonstrated that the prevalence of the utilization of complete PNC was 25.2% [29].

In the present study, respondents who belonged to Brahman/Chhetri (aOR = 4.33, 95% CI: 1.59-11.80) and Janajati (aOR = 4.06, 95% CI: 1.44-11.4) caste/ethnicity were more likely to have complete PNC as compared to Dalits. Consistently, a study conducted in Nepal showed that the Tamang ethnic group was less likely to have had PNC than Brahman/Chhetri (aOR = 0.15, 95% CI: 0.05-0.44) [11]. Similarly, another study in Nepal revealed that women from Janajati were less likely to utilize the maternal services (aOR = 0.75) than women from Brahman/Chhetri [12]. A systematic review and meta-analysis on inequities in the utilization of PNC in the low- and middle-income countries concluded that the use of PNC varies significantly by socioeconomic status of people [30]. The differences in the service utilization might be explained by the differentials in socioeconomic status reflected in the hierarchy of caste/ethnicity persistent in Nepal [31].

The respondents whose husbands were job holders in Nepal were more likely (aOR = 3.6, 95% CI: 1.54-8.66) to have complete PNC as compared to the respondents whose husbands were in foreign employment. Another study in Nepal revealed that mothers whose husbands were involved in the technical field (aOR = 1.7, 95% CI: 1.35-2.17) and labour (aOR = 1.3, 95% CI: 1.10-1.77) were more likely to receive PNC than the respondents whose husbands were involved in agriculture [14]. Another study supports the findings of the current study that women whose husbands were employees of the government or private sectors (aOR = 2.60, 95% CI =1.21-5.98) tended to be more likely to use PNC service than women whose husbands were involved in agriculture and/or labour work [32]. In the current study, however, the husbands were not involved in agriculture but were in foreign employment along with in business, labour, and service. This could be because of the urban setting of the study. Involvement of male is necessary for improved maternal health outcomes in developing countries [33]. Husbands have an important role in the maternity care of their wives [34]. A study conducted in Myanmar revealed that male involvement is more likely (aOR, 2.19; 95% CI, and 1.02-4.69) to result in having complete PNC [29]. In Nepal, husbands' involvement in maternal health care is in giving advice, supporting with household work, and arranging money and transportation for the delivery in a health facility [34]. In addition, the husbands may be the one to accompany wives to health facilities [35]. A husband accompanying his wife is associated with higher likelihood of using PNC from a trained service provider [36]. The involvement of the husband is influenced by his availability, cultural beliefs, and traditions [35].

The present study depicted that respondents who delivered their last child at a private hospital were more likely (aOR = 11.9, 95% CI: 5.52-25.7) to have complete PNC as compared to those who delivered in a government hospital. This finding is supported by the study conducted in Palestine (aOR = 1.8, 95% CI: 1.0-3.4) that women who delivered at a private hospital had a higher likelihood to have PNC [37]. Consistent results were observed in a study in Zambia where women giving birth at a private hospital/clinic had higher odds (aOR 10.08 95% CI 3.35–30.35) of having PNC than at a government hospital (aOR 7.24, 95% CI 4.92–11.84) or government health center/clinic (aOR 7.15 95% CI 4.79–10.66) [38]. PNC counseling and provision of appointment are associated with a higher likelihood of using PNC service [28]. Timeliness and hospitality to patients are greater in private facilities than in public [39]. Women preferred private facilities for maternal health services because of longer consultation time, better interpersonal and communication skills of health care providers, better treatment, more advanced equipment and devices, availability of female obstetricians, and more flexible appointment times [40]. Women who delivered at health facilities rated private facilities to be of higher quality in terms of amenities and interpersonal aspects than public ones [41]. Consistently, interpersonal process was also found of better quality in private health facilities by the women attending health facilities for family planning service [42]. Public facilities are generally used due to financial and physical accessibility [43].

## 5. Strengths and Limitations of the Study

There was a possibility of recall bias as the mothers were asked about the utilization of the service received within last one year prior to the data collection. The findings may not be generalizable in all places as the sample was restricted to four wards of the municipality. Some risk factors have not been measured in this study such as neonatal complication and sociocultural practices during puerperium. However, the study has some strengths as well. The study identifies the status of the utilization of complete PNC in which very limited information is available in community-based studies in Nepal. Secondly, the study shows the status of the utilization of PNC in an urban setting where almost all mothers had institutional delivery, unlike in previous studies of Nepal.

## 6. Conclusion

The utilization of complete PNC is low, although having at least one PNC was high, despite the availability of and accessibility to the service. Caste/ethnicity, occupation of respondents' husband, and type of health facility used for delivery were found to have influenced the utilization. Uptake of complete PNC service may increase by raising awareness on the importance and schedule of PNC. PNC services should be focused on the mothers who belong to a disadvantaged caste/ethnicity and whose husbands are in foreign employment. Involvement of husbands in maternity care, including in PNC service utilization, should be promoted. In addition, improving the quality of the service, particularly in government health facilities, such as interpersonal aspects, timely service, and amenities, may increase the use of PNC service. Implementation research to explore feasibility and sustainability of providing complete PNC by a health worker such as ANM by home visits is needed considering difficult geography and fragile condition of the mother and newborn to travel to a health facility for second and third PNC.

## Figures and Tables

**Table 1:** Sociodemographic characteristics of respondents (*n* = 318).

Variables	Frequency	Percentage
Age of mothers (in years)		
Less than 20	26	8.2
20 to 24	124	39.0
25-29	120	37.7
30 and above	48	15.1
Ethnicity		
Brahman/Chhetri	148	46.5
Janajati	95	29.9
Dalits	75	23.6
Religion		
Hindu	290	91.2
Non-Hindu^∗^	28	8.8
Maternal education		
No schooling	13	4.1
Basic education	49	15.4
Secondary education	189	59.4
Higher education	67	21.1
Maternal occupation		
Household work	167	52.5
Farmer	55	17.3
Service	48	15.1
Business	34	10.7
Labour	14	4.4
Type of family		
Nuclear	173	54.4
Joint	145	45.6
Annual family income (in NPR)		
Less than 250000	120	37.7
250000 to 600000	113	35.6
More than 600000	85	26.7
Occupation of respondent's husband		
Foreign employment	98	30.8
Business	83	26.1
Service (job holder)	81	25.5
Labour	56	17.6
Parity		
One	155	48.7
Two or more	163	51.25

^∗^Buddhist, Christian, and Islam.

**Table 2:** Obstetrical characteristics of respondents (*n* = 318).

Variables	Frequency	Percentage
Had ANC during last pregnancy		
Yes	316	99.4
No	2	0.6
Number of ANC visit (*n* = 316)		
<4 times	37	11.7
≥4 times	279	88.3
Place of delivery of last child		
At health facility	314	98.7
At home	4	1.3
Type of health facility used for delivery (*n* = 314)		
Government hospital	265	84.4
Private hospital	49	15.6
Type of delivery		
Spontaneous vaginal delivery	238	74.8
Assisted vaginal delivery	11	3.5
Caesarean section	69	21.7
Birth attendant at hospital		
Doctor	103	32.8
Staff nurse/ANM	211	67.2
Birth attendant at home (*n* = 4)		
Relative/friend	2	50.0
Staff nurse	1	25.0
No one	1	25.0
Duration of hospital stay after delivery (*n* = 314)		
≤6 hrs.	65	20.7
7-24 hrs.	157	50.0
>24 hrs.	92	29.3
Had complication during childbirth		
Yes	60	18.9
No	258	81.1
Type of the complications^∗∗^ (*n* = 60)		
Prolonged labour	29	48.4
Severe vaginal bleeding	18	30.0
Meconium stained liquor	12	20.0
Retained placenta	5	8.3
Raised blood pressure	5	8.3
Level of awareness on PNC services		
Poor awareness (<5.50)	161	50.6
Good awareness (≥5.50)	157	49.4

^∗∗^Multiple responses.

**Table 3:** Utilization of PNC.

Variables	Frequency	Percentage
Received PNC (*n* = 318)		
Yes	314	98.7
No	4	1.3
Frequency of PNC (*n* = 314)		
Once	205	65.3
Two times	40	12.7
Three times	69	22.0
Level of utilization of PNC		
Partial	245	78.0
Complete	69	22.0
PNC provided by		
Doctors	108	34.3
Staff nurse/ANM	194	61.9
Health assistant	12	3.8
Place for PNC		
Government hospital	268	85.4
Private hospital/clinic	46	14.6

**Table 4:** Factors associated with the utilization of complete PNC.

Variables	Utilization of PNC services		*p* value
	Fully utilized, N. (%)	Partial utilized, N. (%)	
Age groups (in years)			
Less than 30	58 (21.8)	270 (78.2)	0.846
30 and above	11 (22.1)	37 (77.1)	
Ethnicity			
Janajati	23 (24.7)	70 (75.3)	**0.029**
Brahman/Chhetri	38 (32.3)	109 (74.1)	
Dalits	8 (10.8)	66 (89.2)	
Education status			
No schooling	3(23)	10 (77)	
Basic education	8 (17)	38 (83)	0.668
Secondary education	40 (21)	148(79)	
Higher education	18 (27)	49 (73)	
Occupation of respondents			
Household work	38 (23.0)	127(77.0)	
Farmer	10 (18.9)	43 (81.1)	
Service	11 (22.9)	37 (77.1)	0.907
Business	8 (23.5)	26 (76.5)	
Labour	2 (14.3)	12 (85.7)	
Occupation of respondents' husband			
Service	26 (32.9)	53 (67.1)	
Business	18 (21.7)	65 (78.3)	
Labour	9 (16.7)	45 (83.3)	**0.042**
Foreign employment	16 (16.3)	82 (83.7)	
Annual family income (in NPR)			
Less than250000	24 (20.5)	93 (79.5)	
250000 to 600000	35 (22.2)	123(77.8)	0.797
More than 600000	10 (25.6)	29 (74.4)	
Parity			
One	36 (23.2)	119(76.8)	0.597
More than one	33 (20.8)	126 (79.2)	
Number of ANC visit			
<4 times	4 (11.1)	32 (28.1)	0.101
≥4 times	65 (23.4)	213 (76.6)	
Type of facility used for delivery			
Government hospital	38 (14.3)	227 (85.7)	**<0.001**
Private hospital	31 (63.3)	18 (36.7)	
Had complication during childbirth			
Yes	19 (31.7)	41 (68.3)	**0.044**
No	50 (22.6)	204 (80.3)	
Awareness level on PNC services			
Poor	27 (17.2)	130 (82.8)	**0.041**
Good	42 (26.8)	115 (73.2)	

**Table 5:** Odds ratio of factors associated with use of complete PNC.

Variables	Unadjusted OR (95% CI)	Adjusted OR (95% CI)	Adjusted *p* value
Occupation of respondents' husbands			
Service (job holder)	2.51 (1.23-5.12)	**3.49 (1.50-8.13)**	0.004
Business	1.41 (0.67-2.99)	1.69 (0.71-4.00)	0.229
Labour	1.02 (0.41-2.50)	2.11 (0.72-6.18)	0.171
Foreign employment	1	1	
Type of facility used for delivery			
Government hospital	1	1	
Private hospital	10.2 (5.23-20.2)	**11.4 (5.40-24.2)**	<0.001
Caste/ethnicity			
Janajati	2.71 (1.13-6.48)	**2.87 (1.09-7.53)**	0.031
Brahman/Chhetri	2.87 (1.26-6.54)	**3.18 (1.24-8.12)**	0.016
Dalits	1	1	
Awareness level on PNC			
Poor	1	1	
Good	1.75 (1.02-3.03)	1.34 (0.71-2.54)	0.362
Complication during childbirth			
Yes	1.89 (1.01-3.53)	1.25 (0.58-2.67)	0.561
No	1	1	

1 indicates reference category.

## Data Availability

Data will be available from the corresponding author on reasonable request.

## Abbreviations

ANC: Antenatal care
ANM: Auxiliary nurse midwife
aOR: Adjusted odds ratio
CI: Confidence interval
NPR: Nepalese rupees
PNC: Postnatal care.
